# Exocrine Pancreatic Insufficiency Manifesting Before Insulin Dependence in Adult‐Onset Type 1 Diabetes

**DOI:** 10.1002/ccr3.71825

**Published:** 2026-01-26

**Authors:** Panagiotis Pavlou, Shrawan Pandit, Janaka Karalliedde

**Affiliations:** ^1^ Guy's and St Thomas' NHS Foundation Trust London UK; ^2^ King's College London London UK

**Keywords:** autoimmune diabetes, exocrine pancreatic insufficiency, pre‐symptomatic type 1 diabetes, type 1 diabetes

## Abstract

Exocrine pancreatic insufficiency may precede insulin dependence in adult‐onset type 1 diabetes. Unexplained steatorrhea or weight loss in people with diabetes warrants further investigations including imaging of abdomen/pancreas, exocrine pancreatic function and diabetes antibody testing. Early recognition and multidisciplinary care lead to improvements in symptoms and glycemic control.

## Learning Points/Take Home Messages

- Exocrine pancreatic insufficiency as a feature of T1D is commonly overlooked.
- Clinicians should be aware of this association and include T1D in the list of differential diagnoses in patients with pancreatic insufficiency, in the absence of other pancreatic disease and/or lesions.
- Patient education on the natural course of T1D, dietician review/advice, continuous glucose monitoring, advice on sick day rules, education/support for self‐management with insulin if hyperglycaemia is noted should be considered in managing such patients with T1D before insulin dependence.

## Graphical abstract

Exocrine Pancreatic Insufficiency Manifesting Before Insulin Dependence in Adult‐Onset Type 1 Diabetes. Unexplained steatorrhea or weight loss in people with diabetes warrants further investigations including imaging of abdomen/pancreas, exocrine pancreatic function and diabetes antibody testing. Early recognition and multidisciplinary care lead to improvements in symptoms and glycemic control.

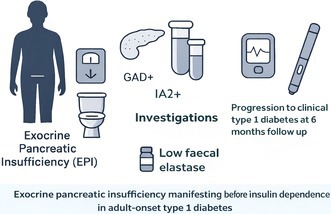

## 1 Background

Exocrine pancreatic insufficiency is reported to occur in approximately one third of patients with T1D; however, it is often overlooked. There is a common misconception that Type 1 Diabetes is a beta cell specific disease; cases such as the one presented challenge this notion, suggesting a diffuse pancreatic disease process. To our knowledge, the presence of exocrine pancreatic insufficiency has only been described in retrospective and cross‐sectional studies in patients with a pre‐existing diagnosis of T1D. The mean duration of diabetes in these studies ranged from 4.9 to 12.5 years; the association between diabetes duration and prevalence of EPI is unclear, with certain publications describing a positive correlation while others found no association [1, 2, 3]. To our knowledge, EPI presenting before insulin dependence in T1D has not been described before in the literature. This case highlights the possibility for exocrine pancreatic insufficiency to clinically manifest before insulin dependence in T1D.

## 2 Case Presentation

A South Asian man in his 30s presented to the gastroenterology clinic with an 8‐month history of weight loss and steatorrhoea. He noted increased frequency of bowel movements and that upon consumption of red meat or spicy food, he had difficult‐to‐flush, oily stools. This was associated with weight loss of 5 kg in 1 year. His weight at the time of the clinic consultation was 70 kg, with a body mass index (BMI) of 27.6 kg/m^2^.

Regarding his past medical history, he had a diagnosis of type 2 diabetes (T2D) made 2 years prior based on routine HbA1c performed in primary care as part of surveillance screening due to history of prediabetes 1 year prior. He also had hypertriglyceridemia and a family history of T2D. He was asymptomatic at the time of diagnosis of T2D. He was commenced on metformin 500 mg twice daily and lifestyle modifications. He was previously diagnosed with non‐alcoholic steatohepatitis and vitamin D deficiency. He did not have any previous history of pancreatitis and only consumed alcohol on rare occasions. The patient was a lifelong non‐smoker and worked as a healthcare professional.

**FIGURE 1:**
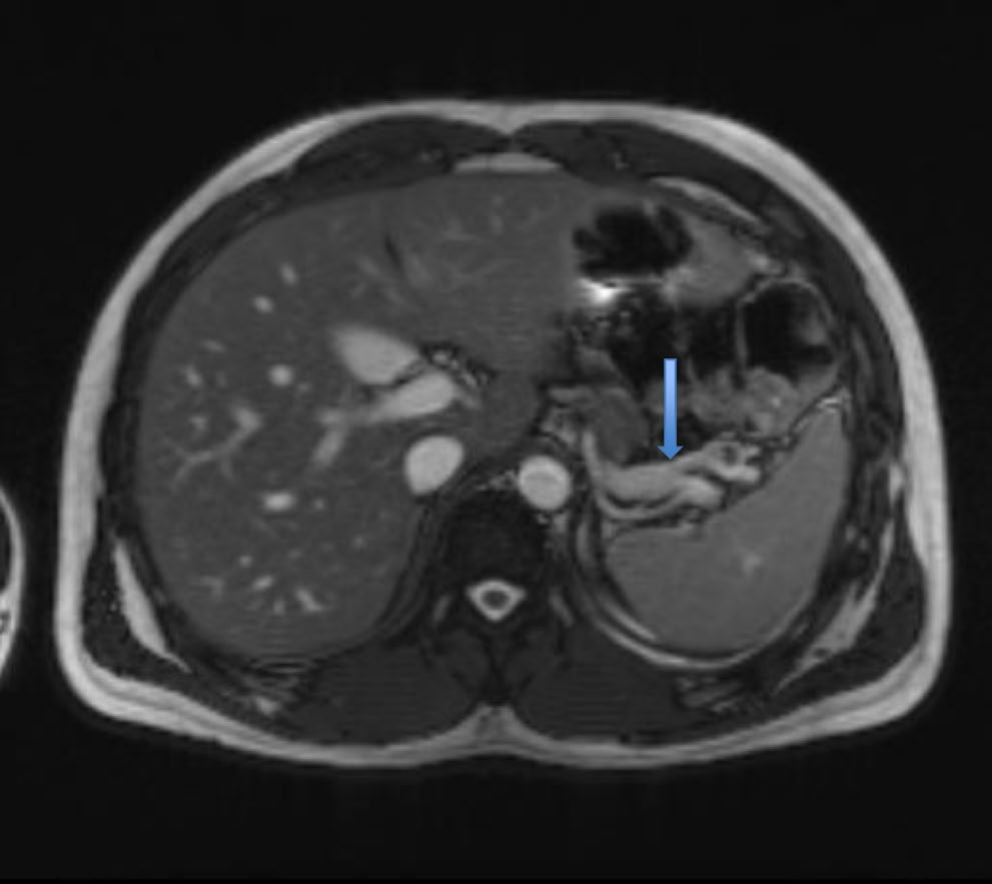
Generalized loss of pancreatic volume is demonstrated (see arrow).

The gastroenterology team performed investigations for exocrine pancreatic insufficiency and faecal elastase was found low on two separate samples. Faecal calprotectin was within the normal range and faecal occult blood test was negative. Liver function, full blood count, serum amylase and lipase, renal and bone profiles were unremarkable. Anti‐tissue transglutaminase antibody was negative and Immunoglobulin G4 (IGG4) levels, which are associated with autoimmune pancreatitis, were within normal range. Magnetic resonance imaging (MRI) scan of the pancreas revealed no pancreatic disease but showed generalised loss of volume (Figure 1). Endoscopic ultrasound of the pancreas showed a hypoechoic parenchyma throughout with no calcification or fibrosis. He was commenced on pancreatin 25,000 IU with meals.

The patient was subsequently referred to the diabetes specialist clinic to help manage his diabetes in the context of exocrine pancreatic insufficiency. Given the association between T1D and EPI, diabetes autoantibody tests and c‐peptide levels were performed. The results were positive for Glutamic Acid Decarboxylase and Islet Antigen 2 antibodies. C peptide concentration was 821 pmol/L with a paired venous blood glucose of 9.2 mmol/L. HbA1c levels were within the diabetes range (> 48 mmol/mol) on three separate samples within the past year. The laboratory results are summarized in Table 1. There were no signs of insulin resistance on physical examination. These results alongside the clinical picture were diagnostic of adult‐onset T1D which is also referred to as latent autoimmune diabetes in adults (LADA) in some publications [4, 5]. Exocrine pancreatic insufficiency had presented prior to insulin dependence. The patient was counseled on the natural course of his condition and the need for insulin therapy in the future. He was placed under appropriate follow‐up for further education and initiation of continuous glucose monitor (CGM).

**TABLE 1:** Biochemistry results.

	Lab reference range	22/11/2023	10/10/2024	23/11/2023	24/05/2024	02/10/2024	10/10/2024	04/12/2024	22/1/2025
HbA1c (mmol/mol) (%)	< 42 mmol/mol < 6%			55 mmol/mol 7.2%	50 mmol/mol 6.7%	50 mmol/mol 6.7%		45 mmol/mol 6.3%	44 mmol/mol 6.2%
C‐peptide (pmol/L)	258–1718 pmol/L							821	
Glucose (mmol/L) (mg/dL)	Fasting < 6.1 mmol/L							9.2	
TSH (mIU/L)	0.3–4.2 mIU/L						1.43		
Anti‐TTG Ab (U/mL)	< 7 U/mL			0.6					
GAD Ab (U/mL)	< 5 U/mL							76	
ZnT8 Ab (U/mL)	< 4 U/mL							< 1	
IA2 Ab (U/mL)	< 7.5 U/mL							9	
Fecal elastase (μg/g)	> 200 μg/g	1.7	1.5						
Faecal calprotectin (μg/g)	0–50 μg/g	< 5					10		
AST (U/L)	5–34 U/L					46			
ALP (U/L)	30–130 U/L					80			
ALT (U/L)	< 55 U/L					34			
Albumin (g/L)	35–50 g/L					50			
Total bilirubin (μmol/L)	< 21 μmol/L					14			
Amylase (U/L)	30–118 U/L			32	23				
Lipase (U/L)	13–60 U/L				5				

Abbreviations: ALP, Alkaline Phosphatase; ALT, Alanine Transaminase; Anti‐TTG, Tissue Transglutaminase Antibody; AST, Aspartate Transaminase; GAD Ab, Glutamic Acid Decarboxylase Antibody; HbA1c, Glycated Hemoglobin A1C; IA2 Ab, Insulinoma Associated Antigen 2 Antibody; TSH, Thyroid stimulating hormone; ZnT8 Ab, Zinc Transporter 8 Antibody.

**FIGURE 2:**
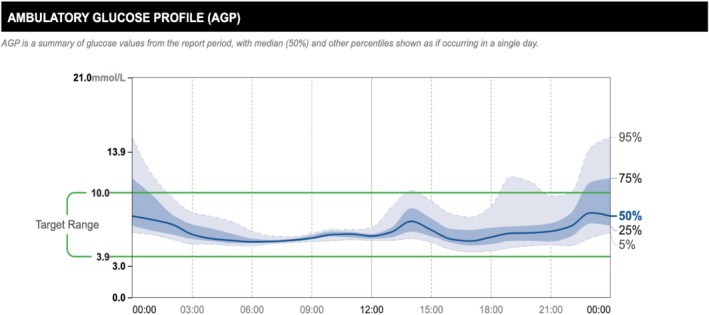
Ambulatory glucose profile (AGP): Two‐week AGP report from continuous glucose monitor at 1 month of follow‐up. Evidence of dysglycaemia in the fasting state and intermittent post‐prandial hyperglycaemia most significant with evening meal, which for the patient was the most carbohydrate‐heavy meal of the day.

**FIGURE 3:**
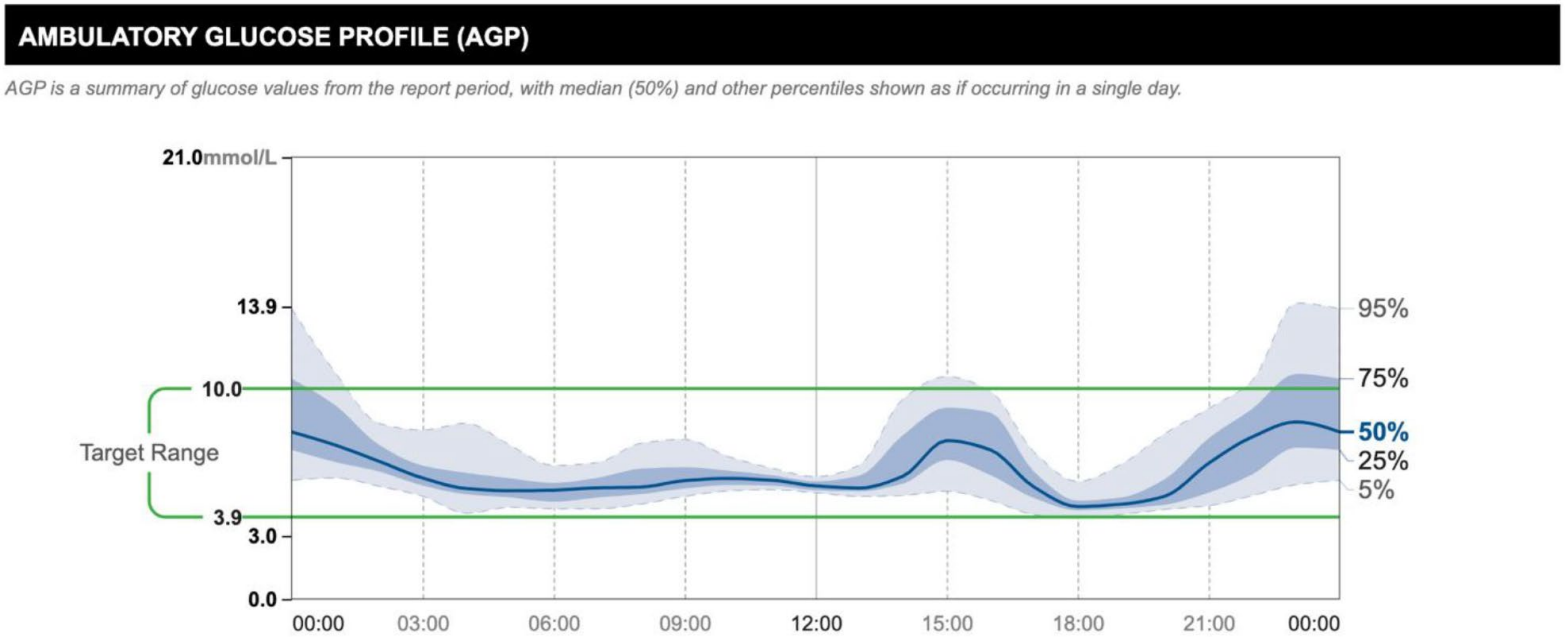
Ambulatory glucose profile (AGP): Two‐week AGP report from continuous glucose monitor at 6 months of follow‐up. Evidence of dysglycaemia in the fasting state and more pronounced post‐prandial hyperglycaemia. At this point, unintentional weight loss was ongoing.

After 6 months of follow‐up under the specialist diabetes clinic, his CGM profile was indicative of intermittent post‐prandial hyperglycaemia and fasting dysglycaemia (see glucose profile in Figures 2 and 3) and he had also experienced unintentional weight loss of 3 kg over the previous 3 months, despite good control of EPI and the associated gastrointestinal symptoms with pancreatin. Prandial insulin aspart was initiated and metformin was stopped. He remains under regular follow‐up with the diabetes clinic and has a designated point of contact for escalation of any concerns.

## 3 Treatment

The gastroenterology team commenced Creon 25,000 IU with each meal to good effect. His initial diabetes management included lifestyle modifications combined with pharmacotherapy with metformin. He was advised to modify diet and undertake regular exercise, approximately 150 min per week. Following his diagnosis of T1D, he was educated on the expected progression of T1D and the future need for insulin therapy. CGM was started in clinic as well as advice and education on sick day rules, and how to self‐administer rapid acting insulin and longer acting basal insulin (if raised glucose levels and blood ketones were noted) provided. The importance of monitoring glucose levels and blood ketone measurement in the setting of inter‐current illness or other stresses was highlighted. All of the above were provided in a multidisciplinary clinic. Follow‐up was arranged in 1 month for further education and surveillance of CGM data.

## 4 Outcome and Follow‐Up

Steatorrhoea and frequency of bowel movements improved with higher doses of pancreatin therapy when pancreatin dose was increased to 50,000 units with meals. Good glycaemic control was maintained on metformin and lifestyle measures, and HbA1c showed improvement. Frank and sensitive communication of the diagnosis and expected progression to insulin ensured patient engagement and vigilance for symptoms of severe insulin deficiency. Following the initial diabetes specialist consultation, follow‐up was arranged in 1 month where continuing education on blood glucose, ketone testing, and review of CGM data took place. At 6 months of follow‐up, he progressed to insulin dependence with clinical T1D manifested with fasting dysglycaemia, post‐prandial hyperglycaemia, and unintentional weight loss. Insulin was well tolerated, and good glycaemic control was achieved. He remains under close follow‐up with the diabetes clinic. Our approach may provide an example of pragmatic and patient‐centred management of T1D prior to insulin dependence, the management of which relies largely on clinical judgment.

## 5 Discussion

Exocrine pancreatic insufficiency is common in diabetes, with literature reports of prevalence 40%–50% [6]. The prevalence has been estimated at 40% in patients with type 1 diabetes and 27% in type 2 diabetes [3].

The diagnosis is made using faecal elastase‐1 (FE‐1) alongside clinical symptoms. FE‐1 is a sensitive and specific test for EPI and correlates well with direct pancreatic function testing [3]. Conversely, exocrine pancreatic disease can also cause diabetes, in the form of Type 3c diabetes. This can be caused by any form of pancreatitis or other pancreatic insult, including conditions such as cystic fibrosis and maturity‐onset of the young with mutations in the carboxyl ester lipase gene (MODY‐CEL) [7, 8], while in a subset of people with chronic pancreatitis, positive islet antibodies can be observed [9]. In our case however, this possibility is ruled out by the absence of any characteristic clinical signs or symptoms of pancreatitis and persistently low amylase and lipase levels. Low amylase and lipase levels have been reported in many studies of EPI in T1D, in keeping with our findings [10].

Existing data suggest that treatment of EPI in patients with diabetes is correlated with improvement in gastrointestinal symptoms, frequency of hypoglycaemia [11] and glucose variability [12]. The pathophysiology of exocrine pancreatic insufficiency in diabetes, however, remains elusive and is likely multifactorial.

Atrophy of the pancreas is common in diabetes and may play a central role in the development of EPI [13]. Insulin has a trophic effect on pancreatic acinar tissue through the insulin‐acinar portal system, and decreased levels could lead to pancreatic atrophy [14]. Moreover, hyperglycaemia inhibits basal and cholecystokinin‐stimulated pancreatic enzyme secretion with an insulin‐independent mechanism and promotes proliferation and activation of pancreatic stellate cells via the protein kinase seC‐p38 mitogen‐activated protein kinase pathway, resulting in pancreatic fibrosis [15, 16]. Islet hormone dysregulation such as chronic glucagon elevation can contribute to atrophy and exocrine dysfunction [17]. Diabetic microangiopathy leads to insufficient perfusion of the exocrine pancreas, which could lead to pancreatic fibrosis, atrophy, and EPI [18]. Diabetes‐induced autonomic neuropathy may lead to impaired enteropathy reflexes and EPI [19, 20]. In addition, autoimmunity against pancreatic cytokeratin and the presence of specific genetic mutations, as single‐base deletion in the variable number of tandem repeats containing exon 11 of the carboxyl ester lipase gene mediate simultaneous damage to exocrine and endocrine tissue [21, 22].

Alterations in signaling protein concentrations in the pancreas may also have a role in pancreatic inflammation and EPI in diabetes. In animal studies, levels of total PKB, p70S6K, 4E‐BP1, ERK1/2, and NF‐kappaB in the pancreas of subjects with diabetes were significantly decreased, while the phosphorylation of p70S6K1, 4E‐BP1, ERK1/2, and protein ubiquitination were increased significantly [23].

The higher prevalence of EPI in T1D is not adequately explained in the literature. It is hypothesized that it is due to more severe insulin deficiency; however, in our case EPI developed before progression to severe insulin deficiency [24].

Interestingly, pancreas volume is reduced in first degree relatives of people with T1D [25] and in stage 1 or 2 (pre‐symptomatic) type 1 diabetes [26]. This was observed in imaging studies in mostly pediatric populations, suggesting alterations to the exocrine pancreas very early in T1D pathogenesis. These findings are in contrast with our case of adult onset T1D with early EPI and atrophy of the pancreas, further highlighting the novelty and relevance of our clinical case report.

Finally, it is worth mentioning Swachman—Diamond syndrome which may present with EPI and T1D. However, in our case this has been ruled out by the age of diagnosis and the absence of family history and other typical clinical features [27, 28].

In our case, magnetic resonance imaging confirmed loss of pancreatic volume, which is consistent with the underlying pathophysiology described in the literature. To our knowledge, no other cases of Type 1 Diabetes first presenting as EPI have been published.

## Author Contributions

**Panagiotis Pavlou:** conceptualization, data curation, investigation, project administration, writing – original draft, writing – review and editing. **Shrawan Pandit:** data curation, writing – original draft. **Janaka Karalliedde:** conceptualization, data curation, supervision, writing – original draft, writing – review and editing.

## Funding

The authors have nothing to report.

## Ethics Statement

Hereby, I, Dr. Panagiotis Pavlou, consciously assure that for this manuscript the following is fulfilled:

1. This material is the authors' own original work, which has not been previously published elsewhere.
2. The paper is not currently being considered for publication elsewhere.
3. The paper reflects the authors' own research and analysis in a truthful and complete manner.
4. The paper properly credits the meaningful contributions of co‐authors and co‐researchers.
5. All sources used are properly disclosed (correct citation).
6. All authors have been personally and actively involved in substantial work leading to the paper and will take public responsibility for its content.

## Consent

Written informed consent from the patient was obtained according to journal guidelines.

## Data Availability

The data that is presented in this manuscript are available from the corresponding author upon reasonable request.
